# Enhanced super‐resolution reconstruction of T1w time‐resolved 4DMRI in low‐contrast tissue using 2‐step hybrid deformable image registration

**DOI:** 10.1002/acm2.12988

**Published:** 2020-09-22

**Authors:** Xingyu Nie, Kirk Huang, Joseph Deasy, Andreas Rimner, Guang Li

**Affiliations:** ^1^ Department of Medical Physics Memorial Sloan Kettering Cancer Center New York NY 10065 USA; ^2^ Department of Radiation Oncology Memorial Sloan Kettering Cancer Center New York NY 10065 USA

**Keywords:** deformable image registration (DIR), radiotherapy, respiratory‐induced tumor motion, super‐resolution image reconstruction, time‐resolved 4DMRI

## Abstract

**Purpose:**

Deformable image registration (DIR) in low‐contrast tissues is often suboptimal because of low visibility of landmarks, low driving‐force to deform, and low penalty for misalignment. We aim to overcome the shortcomings for improved reconstruction of time‐resolved four‐dimensional magnetic resonance imaging (TR‐4DMRI).

**Methods and Materials:**

Super‐resolution TR‐4DMRI reconstruction utilizes DIR to combine high‐resolution (highR:2x2x2mm^3^) breath‐hold (BH) and low‐resolution (lowR:5x5x5mm^3^) free‐breathing (FB) 3D cine (2Hz) images to achieve clinically acceptable spatiotemporal resolution. A 2‐step hybrid DIR approach was developed to segment low‐dynamic‐range (LDR) regions: low‐intensity lungs and high‐intensity “bodyshell” (=body‐lungs) for DIR refinement after conventional DIR. The intensity in LDR regions was renormalized to the full dynamic range (FDR) to enhance local tissue contrast. A T1‐mapped 4D XCAT digital phantom was created, and seven volunteers and five lung cancer patients were scanned with two BH and one 3D cine series per subject to compare the 1‐step conventional and 2‐step hybrid DIR using: (a) the ground truth in the phantom, (b) highR‐BH references, which were used to simulate 3D cine images by down‐sampling and Rayleigh‐noise‐adding, and (c) cross‐verification between two TR‐4DMRI images reconstructed from two BHs. To assess DIR improvement, 8‐17 blood vessel bifurcations were used in volunteers, and lung tumor position, size, and shape were used in phantom and patients, together with the voxel intensity correlation (VIC), structural similarity (SSIM), and cross‐consistency check (CCC).

**Results:**

The 2‐step hybrid DIR improves contrast and DIR accuracy. In volunteers, it improves low‐contrast alignment from 6.5 ± 1.8 mm to 3.3 ± 1.0 mm. In phantom, it improves tumor center of mass alignment (COM = 1.3 ± 0.2 mm) and minimizes DIR directional difference. In patients, it produces almost‐identical tumor COM, size, and shape (dice> 0.85) as the reference. The VIC and SSIM are significantly increased and the number of CCC outliers are reduced by half.

**Conclusion:**

The 2‐step hybrid DIR improves low‐contrast‐tissue alignment and increases lung tumor fidelity. It is recommended to adopt the 2‐step hybrid DIR for TR‐4DMRI reconstruction.

## 1 INTRODUCTION

Patient breathing irregularities are common and may cause suboptimal radiotherapy of lung cancer.^1^, ^2^, ^3^ However, owing to lack of volumetric four‐dimensional (4D) imaging modality, it is difficult to quantify tumor motion variability. The current planning and treatment target either the internal tumor volume (ITV, the entire tumor motion trajectory) or a partial ITV in a gating window, based on respiratory‐correlated (RC) four‐dimensional computed tomography (4DCT), which represents a snapshot of a moving anatomy in a patient's composite single‐breath cycle.^1^, ^2^ In 4DCT, severe breathing irregularities may cause large motion artifacts, affecting the tumor delineation with a variation up to 100% within the cycle.^4^, ^5^ In treatment planning, breathing irregularities have not been accounted for treating a mobile tumor, such as lung cancer,^3^, ^6^ as a 4D volumetric imaging tool is required for multi‐breath motion to evaluate tumor motion variability and treatment uncertainty.

Four‐dimensional magnetic resonance imaging (4DMRI) has been recently studied for MR‐based radiotherapy planning and MR‐guided treatment delivery.^7^, ^8^, ^9^ 4DMRI may appear in four forms: RC‐based 4DMRI, dynamic 2D cine, dynamic 3D cine, and time‐resolved (TR) 4DMRI. RC‐4DMRI, similar to 4DCT, does not provide multi‐breath motion simulation,^10^, ^11^, ^12^, ^13^ dynamic 2D cine MRI does not provide volumetric motion images,^14^, ^15^, ^16^ and dynamic 3D cine cannot provide clinically acceptable spatial resolution.^17^ Facing the challenges to acquire multi‐breath 4DMRI, time‐resolved (TR) 4DMRI technique has been developed using either 2D or 3D cine MRI as guidance for reconstruction based on *a priori* patient‐specific motion model or super‐resolution (SR) mapping using DIR, respectively. The 2D cine‐guided TR‐4DMRI was reconstructed by using a RC‐4DMRI to create a patient‐specific motion model via deformable image registration (DIR) and principal component analysis (PCA).^18^, ^19^ The 3D cine‐guided TR‐4DMRI was reconstructed by mapping the high resolution (highR) from a breath‐hold (BH) image to the low‐resolution (lowR) 3D cine via DIR only.^20^, ^21^ The latter method is independent of RC‐4DMRI as it does not assume periodic motion, and therefore can image irregular organ motion on the fly.

Various DIR algorithms have been developed and the Demons algorithm often outperforms others, with an uncertainty at the image voxel size level.^22^, ^23^, ^24^, ^25^, ^26^ In this algorithm, the deformation force and cost function penalty are higher for high‐contrast tissue, such as the interfaces between the lung and diaphragm. However, it is well‐known that DIR does not perform equally well in low‐contrast tissue, as compared with high‐contrast tissue, thereby tissue contrast becomes a detriment to the image quality of TR‐4DMRI. For low‐contrast tissue, the landmark clarity, deformation force, and misalignment penalty are small, leading to low DIR accuracy. To enhance DIR accuracy, image segmentation,^27^, ^28^, ^29^ motion modeling,^30^, ^31^ as well as correspondence points using scale‐invariant feature transform,^32^ have been incorporated in the DIR process. As new constraints were added to regulate the DIR process, extra uncertainties were also introduced. Although image segmentation has been applied in DIR to enhance DIR accuracy by effectively handling the sliding motion,^27^, ^30^, ^33^ most applications are related to computed tomography (CT) or 4DCT images, whereas little has studied for DIR of thoracic MR and/or 4DMRI images where lung image contrast is much lower than CT/4DCT images.

In this study, a novel hybrid DIR method was introduced to reconstruct TR‐4DMRI image in a two‐step process: Step 1 is the conventional DIR to achieve alignment of high‐contrast tissue and Step 2 is hybrid DIR refinements to improve low‐contrast tissue alignment in the lungs and “bodyshell” (= body ‐ lungs) after automatic lung segmentation and intensity renormalization in each sub‐region of interest (sub‐ROI). Finally, the lungs and bodyshell were put back together for a final tuning at the interface. A T1w‐mapped 4D XCAT digital phantom with a synthesized spherical tumor at low contrast was created. Seven volunteers and five lung cancer patients were scanned under an IRB‐approved protocol: for each subject two high‐resolution BH and a 40s low‐resolution 3D cine FB MR images were acquired. The TR‐4DMRI images from the 1‐step conventional DIR and 2‐step hybrid DIR were compared based on blood vessel bifurcations for volunteers and the tumor position, size and shape for phantom and patients.

## 2 MATERIALS AND METHODS

In the super‐resolution (SR) reconstruction of TR‐4DMRI, a high‐resolution BH image and a low‐resolution 3D cine image series were combined through DIR to map the high‐resolution in the static image to the dynamic low‐resolution images.^20^ An improved Demons DIR algorithm was utilized with a pseudo Demons force to boost deformation range up to 6 cm. The same T1‐weighted (T1w) pulse sequence, acceleration methods and field of view were applied to facilitate the DIR process. The ROI for conventional DIR was defined by the union of the auto‐segmented body contours in two images, while two sub‐ROIs (the lungs and bodyshell) for hybrid DIR were separated by the automatically segmented lung contour. Air bubbles in the abdomen were “removed” by re‐assigning their voxel intensity with the average intensity in the bodyshell before local DIR refinement. The image reconstruction was performed on a workstation (HP Z620, duel Xeon CPU ES‐2620 2GHz and RAM 32GB).

### 2.A Dynamic and static 3D cine MRI image acquisition

Under an IRB‐approved protocol, seven healthy volunteers and five lung cancer patients were recruited and scanned using a 3T MR scanner (Ingenia, Philips Healthcare, Amsterdam, the Netherlands). To accelerate 3D cine scan with a field of view covering the entire lungs and liver, a multi‐shot, turbo field echo sequence was applied with an echo time/repetition time of 1.9/4.2ms and a flip angle of 15°, together with a SENSE factor of 2.5–4 for BH and 6 for FB, partial Fourier factor of 0.8, and the center‐to‐peripheral scanning order (CENTRA). The subjects were in the supine position with both arms up and the image was scanned coronally. The spatial encoding along the slice direction was in the anterior‐posterior direction, the phase encoding was in the right‐left direction, and the readout was in the superior‐inferior direction. For each subject, three T1w MRI sets were scanned: two 12‐20 s BH (BHI at inhalation and BHE at exhalation) and a 40 s FB 3D cine series (80 images). The high‐resolution BH images had a 2 × 2 × 2 mm^3^ voxel size and low‐resolution FB cine images had a 5 × 5 × 5 mm^3^ voxel size at a 2 Hz frame rate.

### 2.B A two‐step hybrid DIR approach

In step one, conventional DIR was performed with normalized voxel intensity (0.0–1.0) in the ROI of the union of body contours in the BH and FB images. The top and bottom 1% voxel intensities in the original 12‐bit image were averaged as the maximum and minimum intensity values for normalization to avoid single‐point spiking noise. A multi‐resolution approach (n = 3: x6, x2, and x1 voxel size of original images) was applied in the Demons DIR process.^21^, ^22^, ^23^ In the coarse resolution level (x6), a pseudo Demons force was applied at a voxel to influence its nearest 124 neighbors in a box of 5 × 5 × 5 voxels with a weight factor following the Gaussian intensity profile (σ = 1).^21^ Therefore, the pseudo Demons force at the coarse alignment step has a long‐distance impact of deformation [2 mm x(5/2) x6 = 30 mm from the voxel] and the function of regulating anatomy integrity upfront, in addition to the Gaussian filter at the end of each iteration. Overall, the conventional DIR can achieve the alignment at high‐contrast tissue, such as the diaphragm.^20^, ^21^

**Fig. 1:**
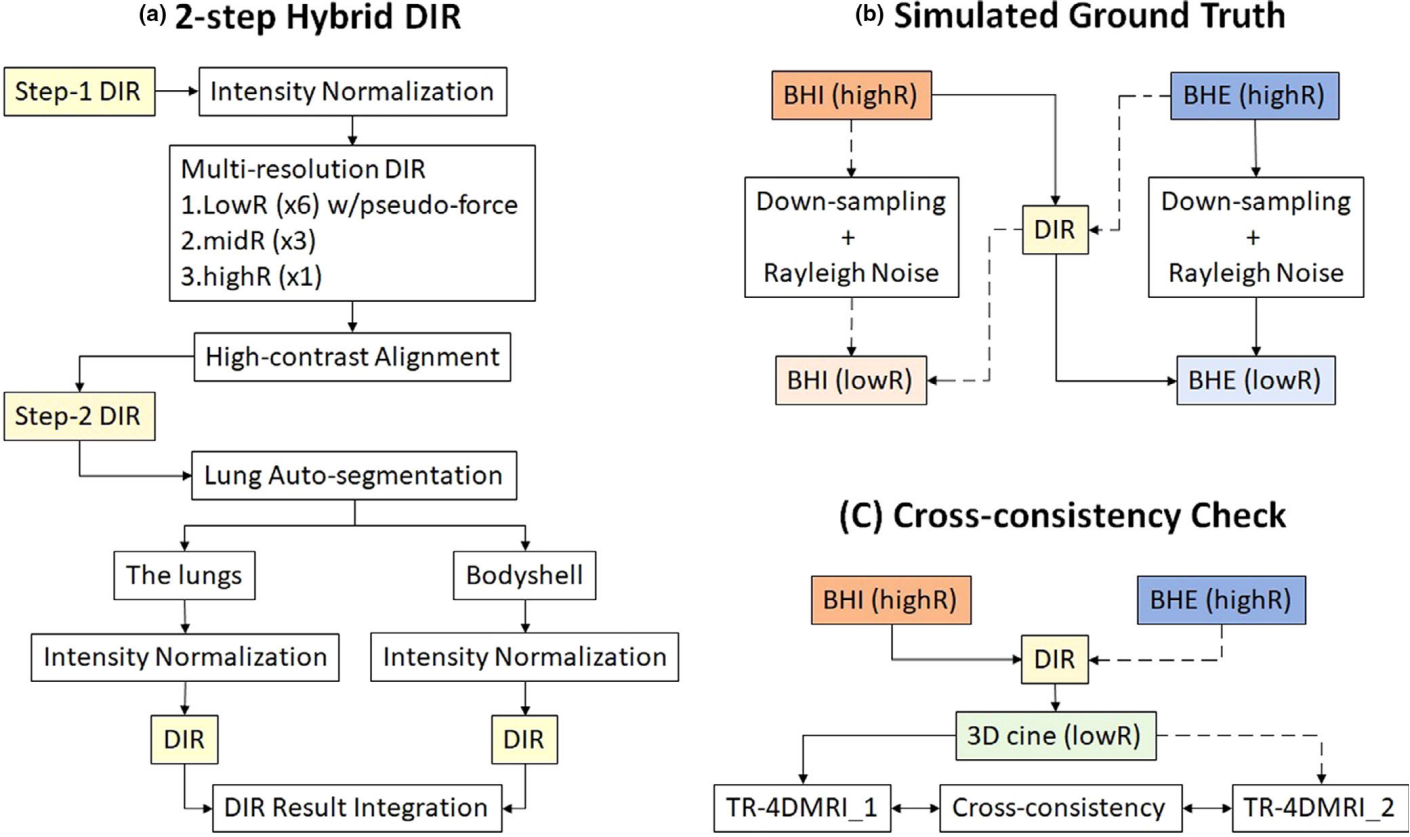
Workflow of 2‐step hybrid deformable image registration (DIR) approach (a), simulated ground truth (b), and cross‐consistency check (CCC) (C). (a) The 2‐step hybrid DIR includes conventional multi‐resolution DIR with pseudo demons force to achieve high‐contrast tissue alignment and two separated DIR after lung auto‐segmentation with the optimized contrast to refine low‐contrast tissue alignment. The final DIR result was obtained by summing up the displacement vector fields (DVFs) from the lungs and bodyshell, applying to the moving image, and performing a final finetuning DIR on the combined image. (b) The simulated ground truth is the original high‐resolution (highR) image of the simulated low‐resolution (LowR) 3D cine as the reference. (c) The CCC compares two 4DMRI images reconstructed from two‐opposite BH images in the inhalation (BHI) and exhalation (BHE).

In step two, the lung in the deformed BH image was automatically segmented and directly mapped to the FB image, serving as the boundary of two sub‐ROIs: the lungs with low‐dynamic range (LDR) display at lower intensity and bodyshell (LDR at higher intensity) in the histogram. In the bodyshell, air bubbles in the bowels were automatically identified and filled with the mean voxel intensity. The LDR in each of the two sub‐ROIs were renormalized (0–1) to utilize the full dynamic range (FDR) of display grayscale (8 bits: 0–255) for contrast enhancement, followed by two separate DIR refinements. Utilizing the FDR for the lungs and bodyshell also amplified the contrast of the LDR tissues. A final finetuning DIR was performed to the integrated image after summing up the two DVFs. The 2‐step hybrid workflow is shown in [Fig. 1(a)]. In the 2‐step hybrid DIR, step 1 may stop early as long as the high‐contrast tissue alignment is achieved since the low‐contrast tissue alignment can be achieved in the second step. Namely, the stopping criteria of voxel intensity difference (VID) change was set ∆VID < 10^−4^ for conventional DIR, but ∆VID < 10^−3^ for hybrid DIR. It is worthwhile to mention that the step‐1 result was also the 1‐step DIR results, which were used as a control to demonstrate enhanced DIR in low‐contrast tissue.

### 2.C Digital XCAT phantom as the ground truth for accuracy assessment

A digital 4D XCAT phantom (version 2)^22^ was mapped with T1w MR image content with a voxel size of 2 × 2 × 2 mm^3^ to serve as the ground truth. The diaphragmatic displacements of 2.0‐6.0 cm with an increment of 1.0 cm were created between the images at full inhalation and full exhalation. Both were down‐sampled to 5 × 5 × 5 mm^3^ and added with 2% Rayleigh noises to mimic a FB image. Then, highR‐to‐lowR DIR was performed in the 1‐step and 2‐step methods and the deformed result was compared with the high‐resolution image for accuracy assessment. As a control, two highR‐to‐highR image DIRs were performed. The voxel intensity correlation (VIC) and structural similarity index (SSIM) were used to assess image similarity between the deformed image and the ground truth. The VIC index was defined as^20^:

(1) $$VIC=\frac{cov\left(I_{m},I_{s}\right)}{\sigma_{I_{m}}\cdot \sigma_{I_{s}}}\;and\;VID=\frac{1}{N}\cdot \sum_{i=1}^{N}\left(I_{m}‐I_{s}\right)$$

where *cov* and *σ* are the co‐variance and standard deviation of voxel intensity (I) strings, and N is the total number of voxels in the static (s) and moving (m) images.

The SSIM index was defined as^35^:

(2) $$\mathrm{CCC}=2\times \frac{\mathrm{VIC}\left({\mathrm{DIR}}^{\mathrm{BHI}},{\mathrm{DIR}}^{\mathrm{BHE}}\right)}{\mathrm{VIC}\left({\mathrm{DIR}}^{\mathrm{BHI}},\mathrm{FB}\right)+\mathrm{VIC}\left({\mathrm{DIR}}^{\mathrm{BHE}},\mathrm{FB}\right)}$$

where DIR^BHI^ and DIR^BHE^ represent BHI→MID and BHE→MID (MID = middle of respiration), respectively. Ideally, CCC should be close to unity. In practice, the *CCC ± σ* was used as a threshold to indicate an acceptable image reconstruction; otherwise, the image needs to be scrutinized for further refinement.^21^ The *σ* was the standard deviation from a set of reconstructions that have been deemed acceptable.

### 2.F Evaluation of DIR quality between 1‐step to 2‐step methods

In addition to the validation methods discussed above, two methods were applied to evaluate the displacement vector field (DVF) to detect any unrealistic deformation. One method was the Jacobian approach, which depicts deformation magnitude, volume change direction, and DVF smoothness.^36^ The Jacobian of the DVF was used to evaluate the invertibility of the DVF transformation. If voxels have negative Jacobian determinants, the deformation is locally noninvertible, causing unrealistic folding‐over within organs. The Jacobian determinant of DVF (*U*) at any given voxel point ($\vec{p}$) was calculated using the following equation^37^:

(4) $$\det\left[J\left(\vec{p}+U\left(\vec{p}\right)\right)\right]=\left|\begin{array}{ccc} \frac{\partial U_{x}}{\partial x}+1 & \frac{\partial U_{x}}{\partial y} & \frac{\partial U_{x}}{\partial z}\\ \frac{\partial U_{y}}{\partial x} & \frac{\partial U_{y}}{\partial y}+1 & \frac{\partial U_{y}}{\partial z}\\ \frac{\partial U_{z}}{\partial x} & \frac{\partial U_{z}}{\partial y} & \frac{\partial U_{z}}{\partial z}+1 \end{array}\right|$$

where *U_x_*, *U_y_*, and *U_z_* are components of U at voxel $\vec{p}$. Usually, 1–3% negative Jacobian of the DVF was thought to be acceptable for a DIR.^38^

The second method was to directly apply the Curl operation to the DVF to identify unnatural local circular motions within an organ.^39^ As each point in the DVF can be expressed as a vector: $\vec{d}=u\vec{i}+v\vec{j}+w\vec{k}$, the mathematical equation was expressed as follows:

(5) $$\nabla \times \vec{d}=\left(\frac{\partial w}{\partial y}‐\frac{\partial v}{\partial z}\right)\vec{i}+\left(\frac{\partial u}{\partial z}‐\frac{\partial w}{\partial x}\right)\vec{j}+\left(\frac{\partial v}{\partial x}‐\frac{\partial u}{\partial y}\right)\vec{k}$$

where (*u, v, w*) were the displacement values in the directions of (*x, y, z*) with the unit vector of ($\vec{i}$, $\vec{j}$, $\vec{k}$). Dramatic changes in the Curl operation of the DVF suggest an unrealistic deformation.

For the DIR results, the Jacobian determinant was applied to calculate the minimum Jacobian value and percentage (%) of negative values. The Curl operation was also applied to assess the realisticity of the DVF. A comparison was made between 1‐step and 2‐step DIR, between BHE‐BHI and BHI‐BHE, and between different motion ranges.

## 3 RESULTS

### 3.A Enhanced low‐contrast tissue alignment using the 2‐step hybrid DIR in volunteers

**Fig. 2:**
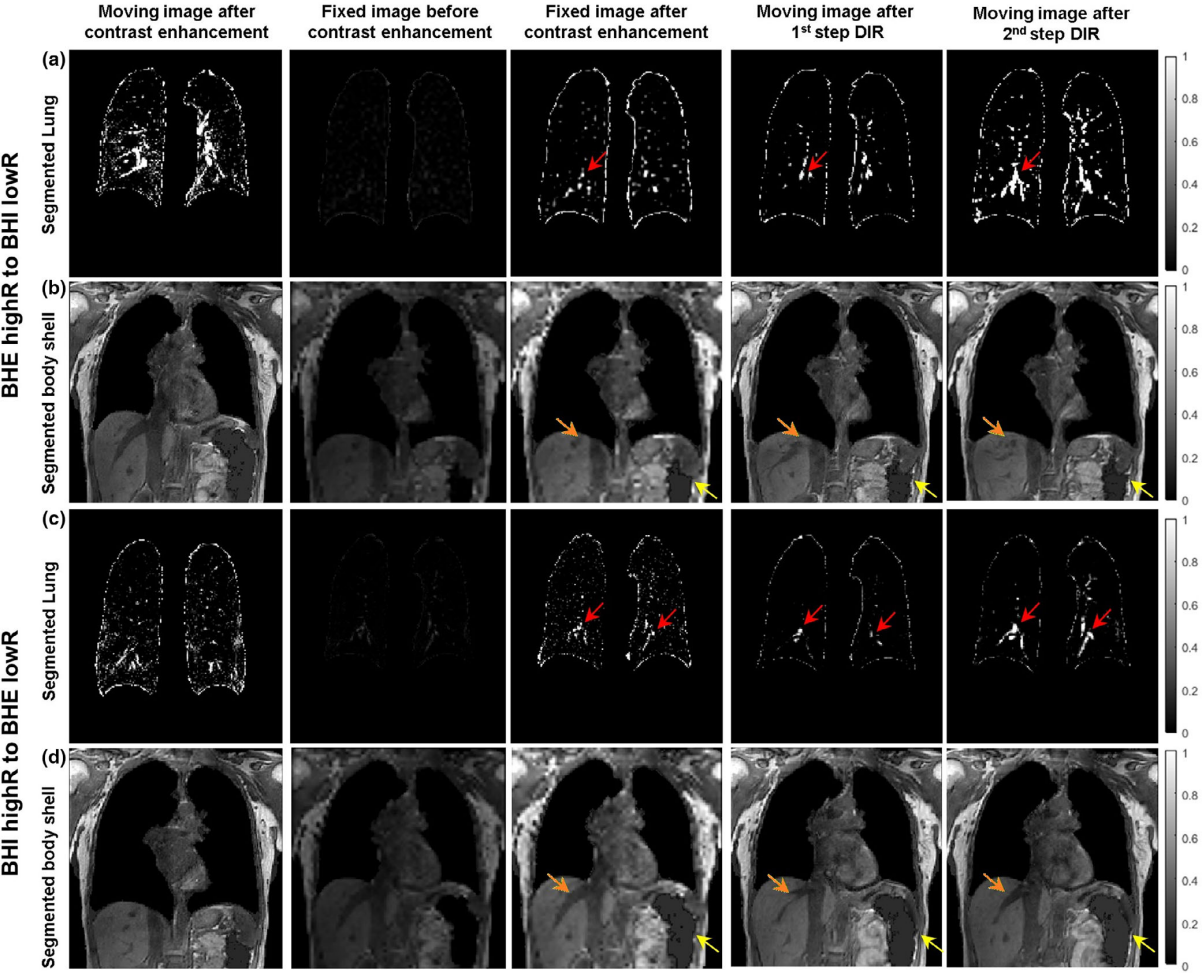
Demonstration of contrast enhancement in both the segmented lungs and bodyshell (the second vs. third columns) and the improvements in lung bifurcation alignment (red arrow) and in liver blood vessels (orange) after the step‐2 DIR (the 3rd to 5th columns). The lung shapes are similar as the alignment is achieved in step‐1 DIR. The air bubble filling (yellow arrow) is achieved (volunteer 5), prior to intensity renormalization for step‐2 DIR. Both directional DIRs are presented

By renormalizing the voxel intensity to the FDR, the lung image contrast is substantially enhanced as illustrated in [Fig. 2(a)], and the bodyshell contrast is also enhanced as shown in [Fig. 2(b)]. The two separated DIR refinements only focus on the alignment of local structures and therefore improve the DIR results. Note that in human subjects, air bubbles in the bowels are common, so the bodyshell needs to be “cleaned” first by filling the air bubbles with the mean tissue intensity before intensity renormalization and DIR refinement.

Table 1 tabulates the comparison between 1‐step and 2‐step DIR results, illustrating the improved accuracy based on the evaluation of 8‐17 low‐contrast bifurcation points in the lungs and liver between the targeted and deformed images. Overall, the average error for 1‐step DIR is 7.0 ± 1.9 mm for BHE‐to‐BHI and 5.9 ± 1.7 mm for BHI‐to‐BHE. By 2‐step DIR, the directional difference diminishes and the errors are reduced to 3.2 ± 1.0 mm (down‐sampled) and 4.0 ± 1.3 mm (down‐sampled and noise‐added). As a control, a 2‐step DIR using two original highR images (BHE and BHI) produces an average error of 2.9 ± 1.0 mm.

**Table 1:** Low‐contrast tissue alignment (in mm) in original and deformed images using conventional and hybrid (2‐step) DIR based on blood vessel bifurcation points in the lung and liver of seven volunteers.

Volunteer Subject	DIR direction	Number of Bifur Points^$^	Original (before DIR)		1‐step DIR (w/o noise)		2‐step DIR^#^ (w/o noise)		2‐step DIR^#^ (w/ noise)		2‐step DIR (hR→hR)	
			Mean	STD	Mean	STD	Mean	STD	Mean	STD	Mean	STD
1	BHE→BHI	13	33.2	3.6	6.1	1.5	3.1	0.9	4.1	0.8	2.6	1.0
2		10	22.3	4.8	5.6	1.3	3.4	0.9	4.0	1.1	3.2	1.2
3		8	46.4	8.7	9.8	3.1	4.0	0.9	4.9	1.0	3.5	1.3
4		13	20.7	4.2	5.7	0.9	3.6	1.2	4.4	1.5	3.0	0.7
5		17	26.0	4.1	5.8	2.6	2.4	1.2	1.9	1.1	1.6	1.0
6		12	34.7	12.8	8.9	2.2	3.1	0.9	4.4	1.6	2.7	1.7
7		8	20.2	4.5	6.9	1.7	4.0	1.0	4.1	1.0	3.7	0.8
**Mean**		**11.6**	**29.1**	**6.1**	**7.0**	**1.9**	**3.3**	**1.0**	**4.0**	**1.2**	**2.9**	**1.1**
1	BHI→BHE	15	37.6	11.4	6.1	2.6	3.3	1.2	4.1	1.4	3.3	1.1
2		10	22.8	5.0	4.6	1.6	2.5	0.7	3.2	1.2	2.1	0.9
3		8	40.1	5.4	7.3	1.3	3.9	0.6	4.9	1.1	4.0	0.5
4		15	20.1	1.7	6.0	1.3	3.5	1.4	4.2	1.5	2.8	1.2
5		15	27.4	4.5	4.3	0.7	2.5	0.8	2.7	0.7	2.2	0.6
6		12	33.5	8.7	6.7	2.3	2.8	1.3	4.4	1.5	2.8	0.8
7		15	18.8	5.3	6.0	1.8	3.2	1.2	4.0	1.7	2.9	1.4
**Mean**		**12.9**	**28.6**	**6.0**	**5.9**	**1.7**	**3.1**	**1.0**	**3.9**	**1.3**	**2.9**	**0.9**

^$^ Equal or more well‐defined bifurcations were found in BHE than BHI, except for subject 5.

^#^ These 2‐step DIRs used down‐sampled lowR image without or with adding 2% Rayleigh noise.

**Fig. 3:**
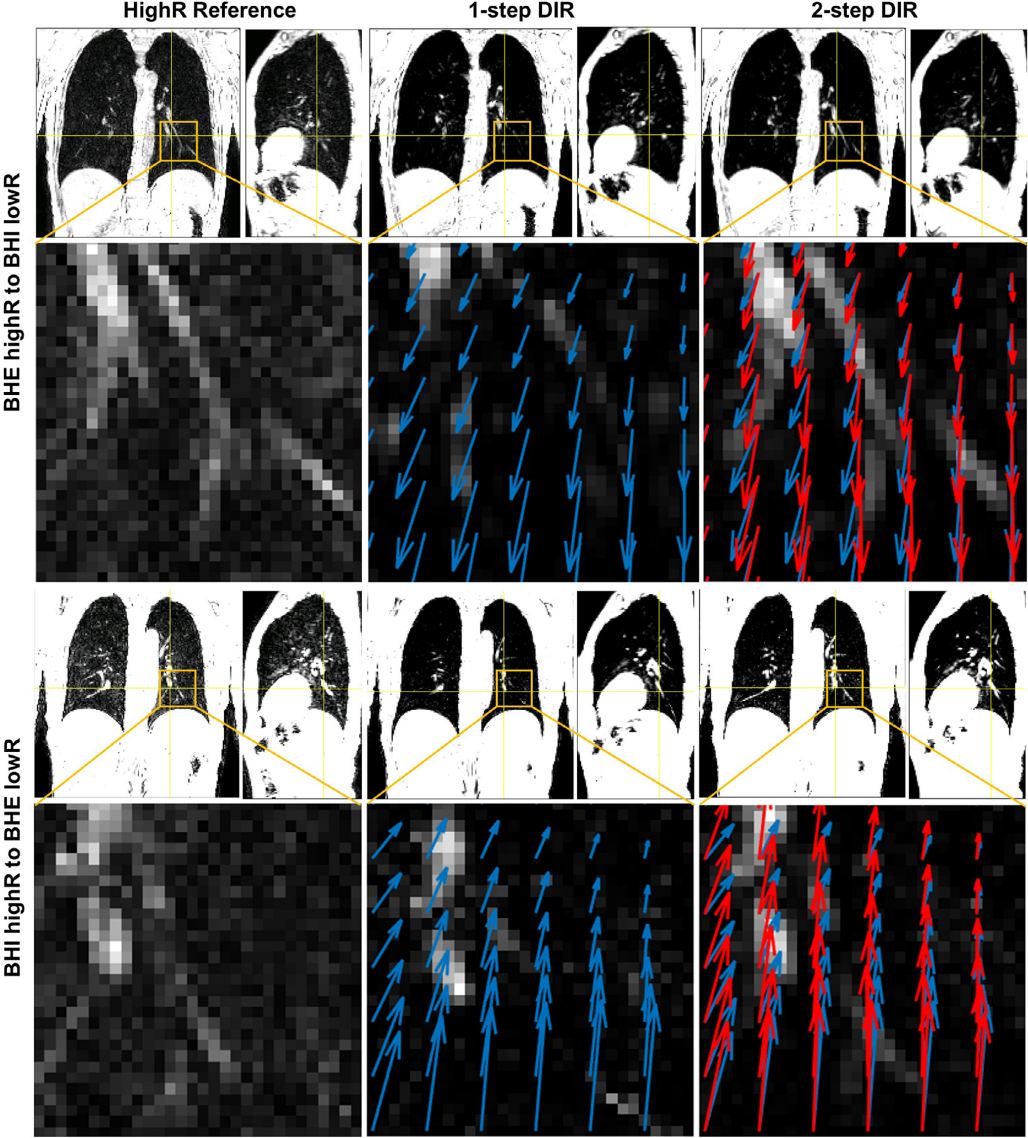
Demonstration of improved lung bifurcation alignment using 2‐step DIR in coronal and sagittal views. The coronal image and DVF around a lung bifurcation point (yellow box) are shown the enlarged inserts, in which the structural similarity between the reference and 2‐step DIR result is shown, differing from 1‐step DIR image. The DVF for 1‐step DIR (blue) and 2‐step DIR (red) is overlaid on the inserts, illustrating the differences in direction and amplitude.

**Fig. 4:**
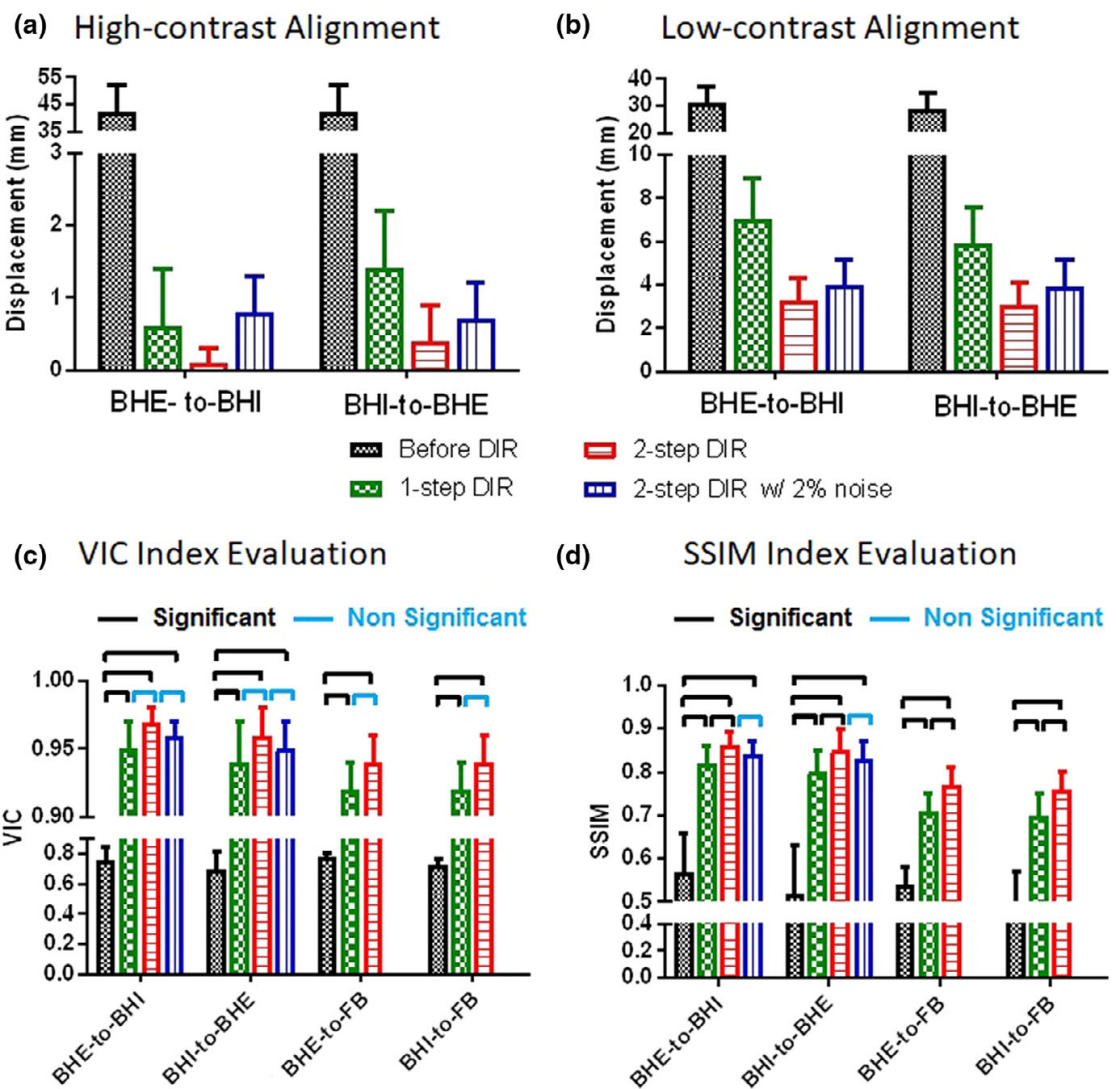
Demonstration of reduced alignment error (in mm) at high‐ and low‐contrast anatomic landmarks (bifurcation structures) and improved voxel intensity correlation (VIC) and structural similarity (SSIM) in the original image and TR‐4DMRI images reconstructed from the 1‐step DIR and 2‐step hybrid DIR methods. (a) high‐contrast alignment, (b) low‐contrast alignment, (c) VIC index and (d) SSIM index. The breath‐hold (BH) images at full inhalation (BHI) and full exhalation (BHE) and free‐breathing (FB) 3D cine images in the middle phase (directly acquired) are applied. The down‐sampling without or with adding 2% Rayleigh noise is used to mimic 3D cine and the Student test is used to assess statistical significance (*P* ≤ 0.05).

Figure 3 illustrates the bifurcation verification after 1‐step and 2‐step DIR refinement against the reference with improved alignment. Figure 4 summarizes the accuracy in low‐ and high‐contrast tissues and the VIC and SSIM indexes, illustrating the improvement by the 2‐step hybrid DIR. In general, the uncertainty is < 1 voxel (2.0 mm) at high‐contrast tissue and < 2 voxels (4.0 mm) at low‐contrast tissue.

### 3.B Better preserved tumor characteristics using the 2‐step hybrid DIR in 4D XCAT phantom

Using the synthetic T1w‐mapped 4D XCAT digital phantom, the spherical tumor moves 1.3–4.0 cm while the diaphragm moves 2.0–6.0 cm. Table 2 tabulates simulated tumor motions and the corresponding registration results on tumor location (center of mass [COM]), size (volume ratio), and shape (Dice index). The 2‐step hybrid DIR refinement substantially improves all the three values, as the ∆COM is at ~ 1 mm level, the volume ratio (V^DIR^/V^REF^) approaches unity, and the Dice index increases to 0.85–0.91, compared to the 1‐step DIR results, especially at large tumor motions. The directional dependency of the DIR is also reduced.

**Table 2:** The tumor alignment in the center of mass (∆COM), volume ratio (V^DIR^/V^REF^), and Dice similarity index of 1‐step and 2‐step DIR results in reference to the target.

DIR Direction*	Diaphragm Motion (cm)	Tumor Motion (cm)	∆COM (mm)		Volume Ratio		Dice Index	
			1‐step DIR	2‐step DIR	1‐step DIR	2‐step DIR	1‐step DIR	2‐step DIR
BHI‐to‐BHE	2.0	1.3	1.3	1.2	0.98	0.97	0.86	0.87
	3.0	2.0	1.6	1.2	0.96	0.99	0.84	0.87
	4.0	2.6	1.7	1.3	0.91	0.98	0.82	0.87
	5.0	3.3	2.2	1.4	0.85	0.99	0.78	0.86
	6.0	4.0	2.5	1.3	0.81	0.99	0.75	0.86
	**Average**		**1.9**	**1.3**	**0.90**	**0.99**	**0.81**	**0.87**
	**STD**		**0.5**	**0.1**	**0.07**	**0.01**	**0.05**	**0.01**
BHE‐to‐BHI	2.0	1.3	0.8	0.8	0.95	1.02	0.91	0.90
	3.0	2.0	0.6	0.8	1.07	0.98	0.89	0.91
	4.0	2.6	1.3	1.0	1.06	0.98	0.86	0.90
	5.0	3.3	2.2	1.3	1.18	0.98	0.79	0.86
	6.0	4.0	1.1	0.9	1.31	0.97	0.75	0.85
	**Average**		**1.2**	**1.0**	**1.12**	**0.98**	**0.84**	**0.88**
	**STD**		**0.6**	**0.2**	**0.13**	**0.02**	**0.07**	**0.03**

Simulated motions in the T1w‐mapped 4D XCAT phantom are 2.0–6.0 cm at the diaphragm and two‐third (1.3–4.0 cm) at the tumor COM. The full inhalation (BHI) and full exhalation (BHE) are used.

* Target images were down‐sampled with added 2% Rayleigh noise to simulate low‐resolution 3D cine images in the 2‐step hybrid DIR for TR‐4DMRI reconstruction.

Bold values are identified as outliers.

**Fig. 5:**
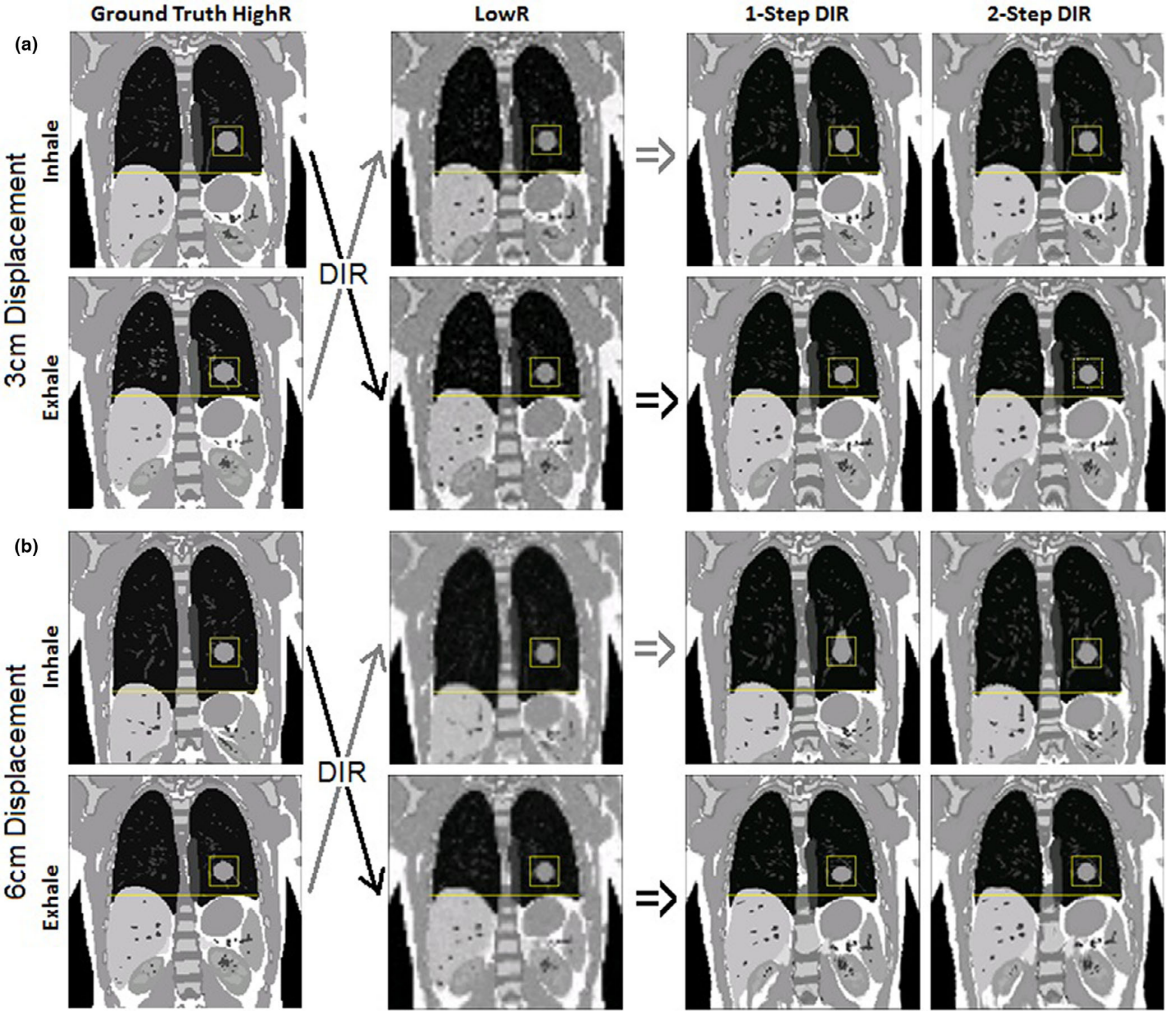
Four examples of reconstructed digital phantom images with a synthesized spherical tumor (Φ = 3.0 cm), compared with the ground truth. The low‐resolution (lowR) images were simulated by down‐sampling the high‐resolution (highR) images with 2% added Rayleigh noise. The 1‐step DIR distorts the tumor while the 2‐step DIR preserves tumor geometry, independent of DIR direction. (a) 3/2‐cm diaphragm/tumor motion, (b) 6/4‐cm diaphragm/tumor motion.

Figure 5 illustrates the visual comparison of the deformed spherical tumor in 1‐step and 2‐step DIR and the ground truth. In 1‐step DIR, the inhalation‐to‐exhalation DIR tends to compress the tumor whereas the exhalation‐to‐inhalation tends to elongate the tumor. The severity of the shape change increases with the increased deformation range from 3.0 to 6.0 cm. However, adding the segmented DIR refinement corrects the distorted tumor volume and shape.

### 3.C Better preserved tumor characteristics using the 2‐step hybrid DIR in lung cancer patients

Table 3 shows that lung tumor position (∆COM), size (volume ratio), and shape (Dice index) are preserved in the TR‐4DMRI images reconstructed using the 1‐step and 2‐step DIR methods in comparison with the highR target reference image. Because the tumor motion in these five patients is < 2 cm, the result is similar to the phantom result with 2cm tumor motion [Table 2 and Fig. 5(a)]. Using the 2‐step hybrid DIR method, the uncertainty of tumor location is ∆COM = 0.4 ± 0.3 mm, the volume ratio is 1.00 ± 0.02, and Dice index is 0.90 ± 0.05.

**Table 3:** Comparison of lung tumor position (center of mass, ∆COM), size (volume ratio), and shape (Dice index) in TR‐4DMRI reconstructed by 1‐step and 2‐step DIR methods with the high‐resolution (highR) target reference image.

Patient	∆COM (mm)		Volume Ratio		Dice Index	
	1‐step DIR	2‐step DIR	1‐step DIR	2‐step DIR	1‐step DIR	2‐step DIR
1	1.6	0.5	0.95	1.00	0.87	0.91
2	0.6	0.1	0.99	1.00	0.96	0.97
3	1.4	0.7	1.00	0.96	0.80	0.85
4	0.3	0.6	0.99	1.01	0.83	0.86
5	0.9	0.3	0.96	1.03	0.88	0.89
**Average**	**0.9**	**0.4**	**0.98**	**1.00**	**0.87**	**0.90**
**STD**	**0.5**	**0.3**	**0.02**	**0.02**	**0.06**	**0.05**

Bold values are identified as outliers.

**Fig. 6:**
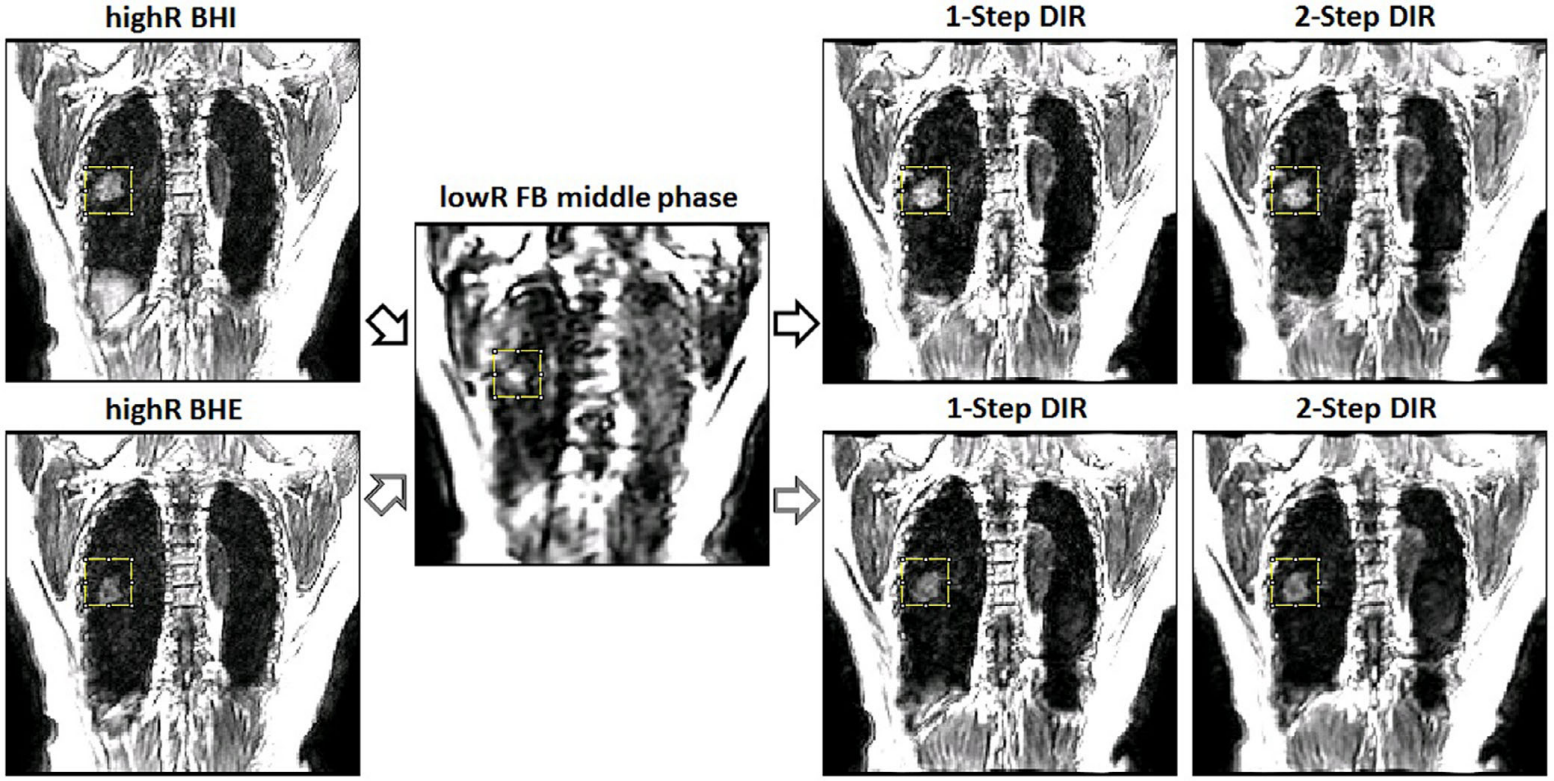
Illustration of the two reconstructed TR‐4DMRI images by deforming two extreme highR‐BH images (BHI at inhalation and BHE at exhalation) to an FB lowR image by 1‐step and 2‐step DIR. The similarity of tumor location, size, and shape in the two reconstructed TR‐4DMRI images is improved from 1‐step DIR to 2‐step DIR.

Figure 6 illustrates an example of cross‐verification of two TR‐4DMRI images of a patient using both BHE and BHI to deform to a FB low‐resolution image for reconstruction. Visually, both reconstructed TR‐4DMRI images are very similar in terms of the tumor location, size, and shape. Quantitative results are included in Table 3 (patient 1). Table 4 tabulates the CCC index for both volunteers and patients using two reconstructed TR‐4DMRI results, together with the VIC index values. After 2‐step DIR refinement, the number of outliers reduced from 6 to 3, which are visually checked for acceptability. The final VIC (0.93 ± 0.02) and SSIM (0.76 ± 0.06) are significantly improved after the segmented DIR refinement.

**Table 4:** The cross‐consistency check (CCC) analysis of the two resultant images using both BH images at inhalation (BHI) and exhalation (BHE) for TR‐4DMRI reconstruction of a middle respiration image (MID).

Subject	1‐step DIR					2‐step DIR				
	VIC (BHE→MID, MID)	VIC (BHI→MID, MID)	VIC (BHE→MID, BHI→MID)	CCC index	within ± σ	VIC (BHE→MID, MID)	VIC (BHI→MID, MID)	VIC (BHE→MID, BHI→MID)	CCC index	within ± σ
V1	0.91	0.92	0.93	1.02	Y	0.94	0.93	0.95	1.02	Y
V2	0.95	0.95	0.96	**1.01**	**N**	0.97	0.97	0.98	1.01	Y
V3	0.91	0.90	0.89	**0.99**	**N**	0.94	0.93	0.92	**0.99**	**N**
V4	0.92	0.92	0.96	1.04	Y	0.92	0.92	0.96	1.04	Y
V5	0.92	0.91	0.93	1.02	Y	0.95	0.94	0.95	1.01	Y
V6	0.94	0.94	0.95	**1.01**	**N**	0.96	0.96	0.96	1.00	Y
V7	0.87	0.89	0.93	1.06	Y	0.89	0.92	0.94	1.03	Y
P1	0.89	0.89	0.97	**1.09**	**N**	0.91	0.91	0.97	**1.07**	**N**
P2	0.91	0.91	0.95	1.04	Y	0.93	0.93	0.95	1.02	Y
P3	0.91	0.91	0.95	1.04	Y	0.94	0.94	0.96	1.03	Y
P4	0.86	0.85	0.94	**1.09**	**N**	0.89	0.89	0.96	**1.08**	**N**
P5	0.88	0.88	0.95	**1.08**	**N**	0.92	0.92	0.96	1.05	Y
Mean				1.04					1.03	
STD (σ)				0.03					0.03	

Out of the 12 cases, the number of outliers (CCC is outside of average ± σ) reduced from six to three cases. The voxel intensity correlation (VIC) index values are provided.

Bold values are identified as outliers.

**Fig. 7:**
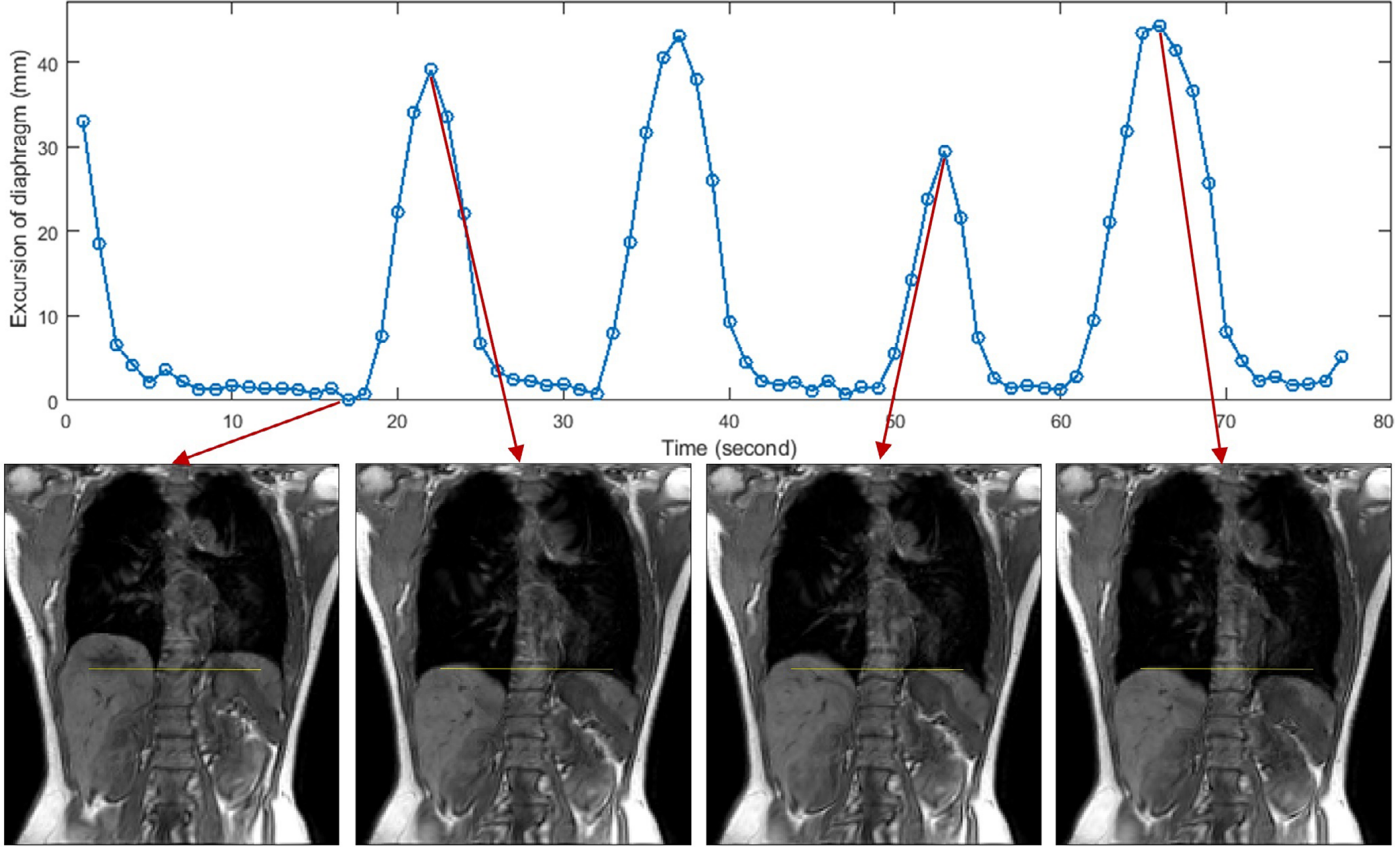
Demonstration of multi‐breath waveform of a representative subject (volunteer 2) with breathing irregularities based on reconstructed TR‐4DMRI. The motion range of the diaphragm is stable in full exhalation phases but variable in full inhalation phases.

Figure 7 depicts an example of multi‐breath TR‐4DMRI images with a respiratory waveform measured on the lung‐diaphragm interface of a subject. The multi‐cycle waveform illustrates breathing irregularities for a relatively well‐behaved breather. This is a unique characteristic of TR‐4DMRI, unlike the respiratory‐correlated 4DMRI with a single breathing cycle, in which breathing irregularities are unavailable but causing binning artifacts.^12^

**Fig. 8:**
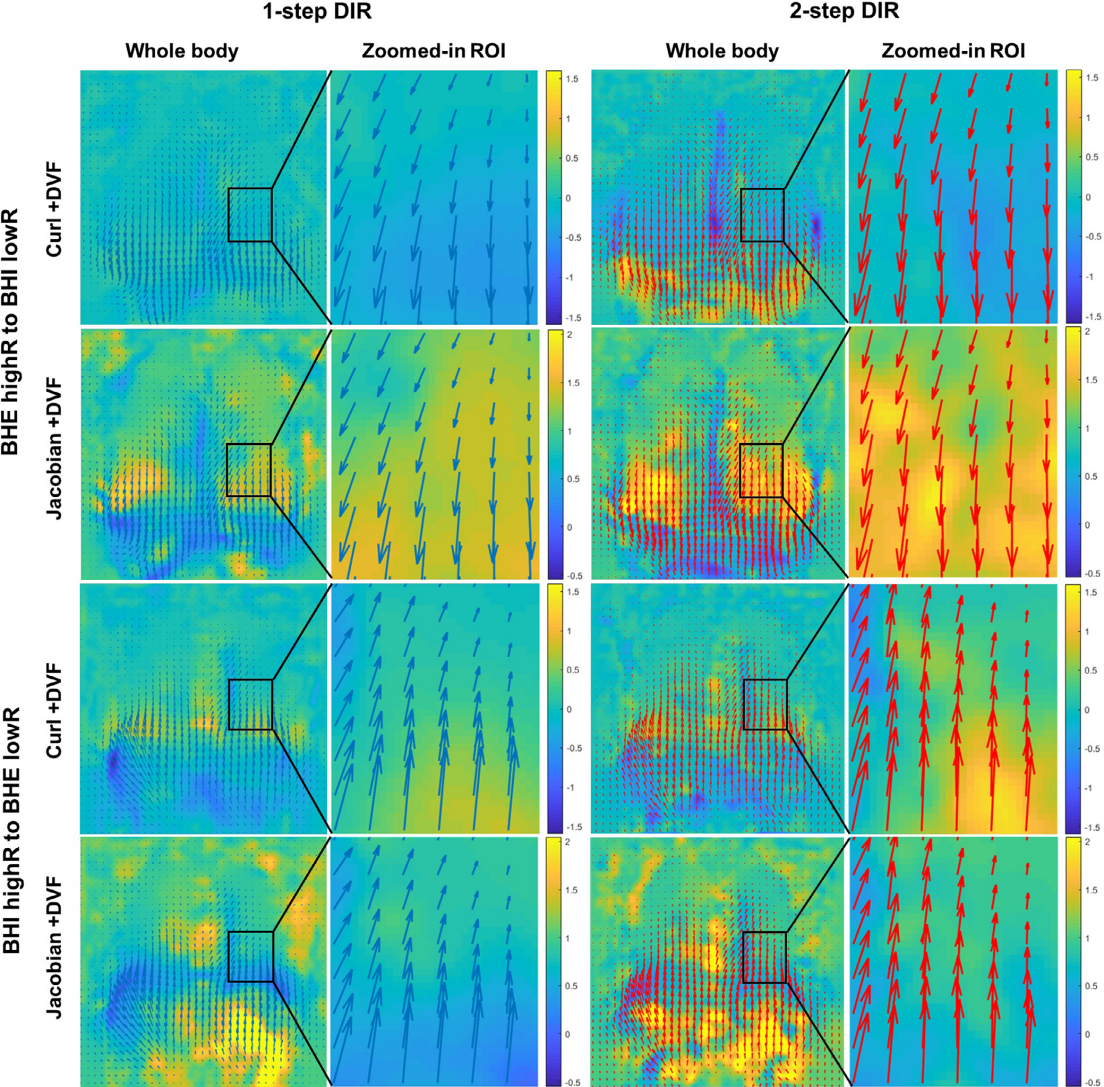
Comparison of the displacement vector field (DVF) overlaid with the vortex map showing Curl values (in mm) and the Jacobian determinant of the DVF (volunteer 4) using 1‐step DIR and 2‐step DIR to deform between breath holds at exhalation (BHE) and at inhalation (BHI), together with a zoomed‐in view on the same ROI as Fig. 3. The arrows indicate the direction and magnitude of displacement vectors at each voxel.

Figure 8 shows the analysis of the Jacobian determinant and Curl operation of the DVF for one of the subjects and Table 5 tabulates the minimal value of Jacobian and % of negative Jacobian. Basically, higher % negative jacobian is observed in 2‐step DIR due to increased iterations, in BHI‐BHE than BHE‐BHI suggesting unequal performance, and in phantom with larger motion range. In this study, the seven volunteers have greater respiratory motion than the five patients.

**Table 5:** The minimum and percentage (%) of negative Jacobian between 1‐step and 2‐step DIR.

Subject	1‐step DIR				2‐step DIR			
	Min Jacobian		% Negative Jacobian		Min Jacobian		% Negative Jacobian	
	BHE‐BHI	BHI‐BHE	BHE‐BHI	BHI‐BHE	BHE‐BHI	BHI‐BHE	BHE‐BHI	BHI‐BHE
D1 (2 cm)	0.00	0.00	0.00	0.00	−0.11	−0.09	0.00	0.00
D2 (3 cm)	0.00	0.00	0.00	0.00	−0.33	−0.34	0.02	0.19
D3 (4 cm)	0.00	−0.16	0.00	0.24	−0.46	−0.38	0.02	1.14
D4 (5 cm)	0.00	−0.55	0.00	1.79	−0.47	−1.09	0.05	3.20
D5 (6 cm)	0.00	−2.03	0.00	2.70	−0.54	−4.86	0.05	4.92
V1	−0.10	−1.26	0.01	1.22	−0.43	−2.40	0.13	2.31
V2	0.00	−0.15	0.00	0.01	−0.42	−0.20	0.01	0.09
V3	−0.69	−1.05	0.03	2.66	−1.14	−1.31	0.38	3.62
V4	0.00	−0.10	0.00	0.01	−0.08	−0.21	0.01	0.04
V5	0.00	−0.61	0.00	0.27	−0.40	−0.90	0.02	0.43
V6	−0.11	−0.16	0.02	0.00	−0.79	−0.31	0.12	0.18
V7	0.00	−0.67	0.00	0.06	−0.14	0.19	0.00	0.19
P1	0.00	0.00	0.00	0.00	0.00	0.00	0.00	0.00
P2	0.00	0.00	0.00	0.00	0.00	0.00	0.00	0.00
P3	0.00	0.00	0.00	0.00	−0.05	−0.04	0.00	0.00
P4	0.00	0.00	0.00	0.00	−0.03	−0.15	0.00	0.00
P5	0.00	0.00	0.00	0.00	0.00	−0.10	0.00	0.00
Average	−0.05	−0.40	0.00	0.53	−0.32	−0.72	0.05	0.96
STD	0.17	0.58	0.01	0.95	0.32	1.25	0.09	1.56

The 2‐step DIR shows slightly lower minimum Jacobian and higher % negative Jacobian compared to the 1‐step DIR, due to more iterations involved in the 2‐step DIR. The mean % negative Jacobian is less than 1%. As the deformation increases, Jacobian negativities increase. The Jacobian analysis also indicates the quality of DIR for BHE‐BHI is higher than BHI‐BHE.

## 4 DISCUSSION

### 4.A Suboptimal alignment of low‐contrast tissue in conventional DIR

Image intensity normalization should be performed before DIR to utilize the full dynamic grayscale range of visualization because the image contrast is affected by the grayscale range of the image in a linear window and level setting. When handling the entire image of a patient, low‐contrast tissue is discriminated due to low‐intensity difference and low‐intensity gradient, which are the two major components of the Demons force for deformation. On the other hand, the misalignment of low‐contrast tissue receives little penalty governed by the VID‐based cost function in DIR optimization. As a result, low‐contrast tissue alignment is often suboptimal and DIR direction‐dependent, resulting in an elongated or compressed tumor (see Fig. 5). Therefore, the low‐contrast tissue alignment in DIR remains a challenge,^24^, ^25^, ^26^, ^40^ and the 2‐step hybrid DIR approach provides an automatic, efficient and effective solution to this known problem.

The conventional DIR with the full image volume aligns well at the high‐contrast tissue, such as the lung‐diaphragm interface, whereas low‐contrast tissue is neglected (biased) due to their small contribution in the shared common stopping criterion. This problem has been recognized and various efforts have been made, including incorporation of segmented points, contours or surfaces into DIR or application of model‐based DIR.^28^, ^30^, ^31^, ^32^, ^41^, ^42^ However, DIR accuracy of these approaches also depends on the accuracy of the segmentation of landmarks, the correspondence of matching points, and the need of interpolation. Therefore, the improvement is usually restricted to specific cases. In contrast, this 2‐step hybrid DIR method takes advantage of high DIR accuracy at high‐contrast interfaces and the readily available auto‐segmentation tools, so that the low‐contrast tissue landmarks in MRI images are enhanced by fully utilizing the FDR of image grayscale while introducing little additional uncertainties in the DIR process.

### 4.B The necessity of the hybrid DIR refinement for low‐contrast tissue alignment

One of the major benefits of incorporating segmentation into DIR is to perform anatomic‐based “piecewise” refinement, rather than a mechanical grid.^43^ For low‐contrast tissue it has a certain relatively narrow intensity range, especially for the lung that differs from other tissues inside the bodyshell. In T1w MR image of the lung, the contrast between lung tissue and blood vessel image is low comparing with that of lung‐bronchial tree in CT. Therefore, to align the lung tissue or tumor, additional image processing is needed, as shown in Fig. 2. In the abdomen region, the contrast in MR is much higher than CT, but still belongs to low‐contrast LDR tissue compared to the lung‐diaphragm interface. Therefore, a boost in tissue contrast is helpful for image alignment. With the automatic lung segmentation tools,^44^, ^45^ the LDR of the lung (usually 0–80) is visualized in the FDR (8 bits: 0–255), leading to enhanced contrast, as shown in [Fig. 2(a)]. Similarly, the intensity range for remaining non‐lung‐tissue, excluding air bubbles in the bowel, can also be displayed in FDR for contrast enhancement. After intensity renormalization, low‐contrast tissue becomes dominant within each of the two sub‐ROIs, while high‐contrast lung/soft tissue interface is eliminated (Fig. 2). Therefore, the low‐contrast tissue alignment is enhanced in step‐2 DIR refinement without the interference from the high‐contrast tissue interfaces.

Unlike conventional piecewise DIR refinement that separates tissue in grids containing both high‐ and low‐contrast tissues, the step‐2 hybrid DIR refinement separates the low‐contrast tissue based on the intensity range, excluding the impact from high‐contrast region. Therefore, the step‐2 DIR refinement reduces the average uncertainty of TR‐4DMRI image reconstruction from 7 to 3.3 mm, close to that in highR‐to‐highR DIR (2.9 mm), based on vessel bifurcation structures as shown in Tables 1, 2, 3. The VIC and SSIM also show improvement in similarity to the reference image after the hybrid DIR (see Fig. 4).

Although extra steps are added in the DIR process, the overall performance remained at a similar level because the time for finetuning in the 1‐step DIR was replaced by step‐2 hybrid DIR refinement. When the stopping criterion is lifted in the first step from ∆VID < 10^−4^ to < 10^−3^, sufficiently accurate high‐contrast tissue alignment has been achieved. Note that the automatic image segmentation time is negligible. Overall, the TR‐4DMRI reconstruction using the 1‐step conventional DIR lasts 10 m on average, whereas the new 2‐step hybrid DIR takes 13 m and there is room for further improvement.

### 4.C Image quality assessment for TR‐4DMRI reconstruction

To reconstruct the SR‐based TR‐4DMRI image, two images with different spatial resolutions are involved, differing from a conventional DIR application. The simulated ground truth allows to assess DIR accuracy [Fig. 1(b)]. The highR‐to‐highR DIR results (shown in Table 1) indicate that the best alignment on the same set of low‐contrast blood vessel bifurcations is 2.9 mm, which is slightly less than 1.5 voxels. Whereas the highR‐to‐lowR DIR has achieved 3.3 mm with down‐sampling only and 4.0 mm with down‐sampling and addition of 2% Rayleigh noise. Therefore, the highR‐to‐lowR DIR is almost as reliable as highR‐to‐highR DIR, with sub‐mm accuracy difference.

Using two high‐resolution BH images to register a series of lowR 3D cine FB images for TR‐4DMRI reconstruction sounds redundant, but it does serve as a cross‐verification mechanism to automatically check the image quality of the reconstructed TR‐4DMRI using the CCC index [Fig. 1(c)]. This is valuable to verify the reconstruction quality as a QA tool to identify likely troubled DIR reconstruction for a further visual check. If the CCC outliers (outside of mean ± σ) passed the visual check, then others are likely to be acceptable. We previously reported that the CCC index successfully identified inferior DIR in the reconstruction, which was then improved by further refinement of the DIR result.^21^ In this study, we have illustrated the enhanced DIR result using the 2‐step hybrid DIR refinement and reduced number of outliers in half, as shown in Table 4. In fact, the outliers are visually checked, and no obvious DIR misalignment is identified, suggesting that all reconstruction results are acceptable. The CCC index could serve as a QA tool to raise a red flag for a potential reconstruction problem in future clinical use.

The degree of unrealistic deformation in the reconstructed TR‐4DMRI images are similar between 1‐step and 2‐step DIR, although the 2‐step DIR results in slightly higher % negative Jacobian, likely due to more optimization iterations.^38^ Both are in an acceptable range. From the phantom results, it is obvious that the larger the motion, the more unrealistic deformation would occur. It is interesting to point out that the DIR performance between BHE and BHI is direction‐dependent: BHE‐BHI is more reliable than BHI‐BHE. In addition, the quality of BHE is slightly higher as equal or more bifurcation points were identified in BHE than BHI, except for subject 5 (Table 1). Therefore, it is recommended to use BHE (or at mid‐respiration but closer to BHE) as the highR image for TR‐4DMRI reconstruction.

### 4.D Advantages and limitations of hybrid DIR reconstruction of TR‐4DMRI

Adding automatic lung segmentation into the DIR process allows to separate the tissue based on the intensity range in the histogram, so that renormalization will expand the LDR to the FDR. Therefore, the low contrast of organ texture in the MRI images is enhanced and becomes dominant in this 2‐step hybrid DIR refinement. It is worthwhile to mention that the conventional DIR is important to achieve high‐contrast tissue alignment, ready for the hybrid DIR refinement on the voxels inside the sub‐ROIs. Moreover, the accuracy of DIR refinement is not dependent on that of lung contouring at the high‐contrast edge; but determined by low‐contrast native landmarks within or outside the lung contour. Guided by the enhanced landmarks, the step‐2 hybrid DIR can finetune soft tissue alignment, so that the dependency of DIR direction can be minimized, indicated by close to unity CCC index (Table 4) as well as the reduced alignment differences (Tables 1 and 2).

The highR‐to‐highR DIR has achieved 1.5‐voxel (2.9 mm) uncertainty, which sets the limit for the current DIR‐based reconstruction technique. However, when the image resolution and quality are improved by incorporating new MRI techniques, such as compressed sensing (CS), the SR‐based TR‐4DMRI reconstruction can be further improved. In addition, improvement of the lowR 3D cine image quality will help to make the reconstruction approach to the limit. By applying the CS‐based MR acquisition and reconstruction, the spatial resolution of the 3D cine in FB can increase to 4 × 4 × 4 mm^3^ from 5 × 5 × 5 mm^3^, whereas the time for BH can be reduced from 20 s to 10 s, facilitating clinical implementation. To further improve the DIR performance, parallel computing using graphics processing unit (GPU), is recommended because it reduces the reconstruction time by 1–2 orders of magnitudes, from minutes to seconds.^46^, ^47^ Therefore, it is expected that the image quality and performance of TR‐4DMRI reconstruction can be further improved.

Finally, a linear window/level setting is applied in this study to illustrate the feasibility and effectiveness of the 2‐step hybrid DIR reconstruction of TR‐4DMRI. However, a nonlinear lookup table that is optimized to the histogram of the ROI can be introduced for further contrast enhancement,^48^ and therefore the DIR accuracy can be further improved for TR‐4DMRI reconstruction.

## 5 CONCLUSION

This study demonstrated that a novel 2‐step hybrid DIR approach is effective to improve image alignment in low‐contrast tissues while maintaining good alignment within high‐contrast tissue using a digital phantom (with 2–6 cm motions), in seven healthy volunteers, and five lung cancer patients. The lung tumor fidelity in the reconstructed TR‐4DMRI image is quantified as ∆COM < 1.0 mm, <3% volume variation, and >0.85 dice index. Substantial improvement in low‐contrast tissue has been achieved in the reconstruction of the super‐resolution TR‐4DMRI image.

## Conflict of Interest

Memorial Sloan Kettering Cancer Center has a master research agreement with Philips Healthcare. This work was presented in the AAPM annual meeting in Nashville, TN in July 2018.
